# Testing how different narrative perspectives achieve communication objectives and goals in online natural science videos

**DOI:** 10.1371/journal.pone.0257866

**Published:** 2021-10-13

**Authors:** Selina A. Ruzi, Nicole M. Lee, Adrian A. Smith

**Affiliations:** 1 Department of Applied Ecology, North Carolina State University, Raleigh, North Carolina, United States of America; 2 School of Social & Behavioral Sciences, Arizona State University—West Campus, Glendale, Arizona, United States of America; 3 North Carolina Museum of Natural Sciences, Research & Collections, Raleigh, North Carolina, United States of America; 4 Department of Biological Sciences, North Carolina State University, Raleigh, North Carolina, United States of America; University of Milan, ITALY

## Abstract

Communication of science through online media has become a primary means of disseminating and connecting science with a public audience. However, online media can come in many forms and stories of scientific discovery can be told by many individuals. We tested whether the relationship of a spokesperson to the science story being told (i.e., the narrative perspective) influences how people react and respond to online science media. We created five video stimuli that fell into three treatments: a scientist presenting their own research (male or female), a third-party summarizing research (male or female), and an infographic-like video with no on-screen presenter. Each of these videos presented the same fabricated science story about the discovery of a new ant species (Formicidae). We used Qualtrics to administer and obtain survey responses from 515 participants (~100 per video). Participants were randomly assigned to one of the videos and after viewing the stimulus answered questions assessing their perceptions of the video (trustworthiness and enjoyment), the spokesperson (trustworthiness and competence), scientists in general (competence and warmth), and attitudes towards the research topic and funding. Participants were also asked to recall what they had seen and heard. We determined that when participants watched a video in which a scientist presented their own research, participants perceived the spokesperson as having more expertise than a third-party presenter, and as more trustworthy and having more expertise than the no-spokesperson stimuli. Viewing a scientist presenting their own work also humanized the research, with participants more often including a person in their answer to the recall question. Overall, manipulating the narrative perspective of the source of a single online video communication effort is effective at impacting immediate objective outcomes related to spokesperson perceptions, but whether those objectives can positively influence long-term goals requires more investigation.

## Introduction

Online media has become a primary means through which public audiences connect with science (e.g., [1]). Online, science media originates from many producers, from traditional news-producing institutions (journalism/news, universities, science societies), non-traditional sources like online personalities (science communicators and YouTubers), and even apparently author-less presentations in the form of memes, infographics, or animations (e.g., [1–7]). Increasingly, research scientists are creating online representations of both themselves and their work for public audiences in forms ranging from institutional websites to personal/professional social media feeds. How and if these self-representations of science are perceived differently from other sources is only beginning to be experimentally tested but understanding its potential impact has relevance to our collective understanding of and recommendations for communicating science.

Public audiences tend to choose to listen to, and believe, science stories presented by sources they like [8] and trust [9]. The vast majority of people cannot name and do not personally know a living scientist [10], thus, perceptions of scientists are largely formed through media depictions [11] which have led to a stereotypical perception of scientists as being cold and aloof [12, 13]. Best practice recommendations for science communication encourage scientists to strategically counter this stereotype by emphasizing communication objectives like demonstrating warmth and corresponding trustworthiness, showing accessibility and relatability, and fostering two-way interactions [14]. Social media platforms where dialogue, expressions of users’ individual personalities, and “selfie” images dominate are spaces in which many of these objectives can be achieved. These platforms present opportunities for highly individuated depictions of research scientists, and opportunities to counter stereotypical perceptions of who scientists are [15, 16]. While many social media platforms exist, they differ in what forms of content are most common from micro-blogging on Twitter (e.g. [17–19]), fan pages and mixed media on Facebook [19, 20], images and short video on Instagram (e.g. [15, 19–21]), and video on TikTok (e.g. [21]) and YouTube [19, 22]. Arguably, social media platforms that favor user-generated video, with users on-screen delivering their content, present the highest fidelity opportunities for public audiences to meet a scientist and for scientist-users to present highly individuated depictions of themselves and their work. These practices lend themselves to first-person narratives, which some research suggests can increase audience-source identification (e.g., [23]).

User-generated video has been a dominant form of content on popular online media platforms for decades, from the original *Broadcast Yourself* slogan of YouTube to selfie-style content of TikTok. Correspondingly, these video-focused platforms have increasingly become recognized and evaluated as popular media for effective science communication [21, 24–26]. Many studies of this type of media assess characteristics of successful YouTube content by evaluating previously published videos from established and popular channels (e.g., [5, 7, 27]). While a few recent studies have included comparisons of videos presented by scientists versus science YouTube presenters [28, 29], controlled experiments that are designed to test the effectiveness of scientists-as-presenters telling first-person narratives in online video compared to other narrative perspectives have yet to be done. In this study, we attempt to address this experimental gap.

### Literature review

#### Strategic communication and source effects

The strategic communication framework recognizes that effective communication stems from a hierarchical set of communication strategies and tactics used to achieve objectives, and goals [30–34]. Objectives are immediate outcomes of communication and contribute to the overall long-term goal [30, 34–36]. Some examples of potential science communication objectives include informing audiences about science, building trust, and defending scientific results [30, 31, 35]. However, some objectives are more effective at influencing long-term goals of science communication than others. For example, effective communication often stems from factors such as trustworthiness, warmth, and audience engagement rather than an objective of informing [37]. These objectives of building trust or sharing values can be especially important for more change-oriented goals (e.g., [38]).

Goals are ultimate, long-term desired outcomes [33, 34, 36] shaped by communication. These include concrete scenarios such as increasing science’s influence in policy making, personal decision making, and funding support for research [33]. Additionally, goals can include more nebulous conditions such as science being more culturally valued. Understanding what the goal of the communication effort is helps to determine what objective to focus on and, in turn, what tactic to choose.

A strategy is the big-picture approach or plan a communicator employs to achieve their desired outcomes. Tactics are the specific decisions on format, venue, style, or even the content of communication messages that carryout that strategy. Communication choices are often based on the individual’s efficacy beliefs about specific tactics and their beliefs about their own skills as communicators (e.g., [32, 39]). Even simple and subtle tactical differences might influence how well core communication objectives are achieved. In fact, a great deal of existing research has focused on how the source or spokesperson presenting a message can influence audience perceptions (e.g., [40–42]). Such research has examined variables including a spokesperson or source’s role or title [41], attractiveness [43], and gender [44]. Related scholarship has explored source effects based on narrative perspective or how a source is related to the information at hand, meaning whether the narrative is told in the first- or third-person. Most commonly studied in health contexts, past findings are mixed and appear to depend on the specific person presenting a narrative, the context, and the individual outcomes of interest. For instance, first-person point of view was found to increase reader-protagonist identification in a health narrative about diabetes [23] but did not influence risk perceptions related to HPV [45]. Although there is existing research on narrative perspective, due to the mixed results and difference in context, more research is needed to understand the influence of a spokesperson’s relationship to scientific research in the context of science narratives. Specifically, are scientists more or less effective when it comes to communicating their own research compared to a third-person narrative about their findings?

The elaboration likelihood model (ELM) posits that messages can be persuasive when thinking is high (central route processing) or low (peripheral route processing) but that the factors that lead to persuasion depend on the route [46]. The model suggests expert sources and credibility cues such as a source’s title can serve as heuristics in peripheral route processing. This may be particularly relevant to the communication of basic science, especially for topics with low personal relevance for the audience, which leads to peripheral route processing. Knowing a scientist is presenting their own work–and therefore has firsthand knowledge on the topic–may act as a heuristic that increases credibility and persuasiveness.

#### Scientists as communicators

Understanding scientists’ effectiveness as spokespeople for their own work is important as they are increasingly expected to engage in public outreach activities [35, 47–49]. A large-scale survey of US-based scientists revealed that nearly all (98%) scientists talk to citizens about science and research, with 51% having experience talking with reporters about their research, and 47% using social media to talk about science [49]. Similarly, US-based scientific societies report their professional memberships voicing an increased demand for science communication and public engagement opportunities [50]. However, many scientists have a narrow view on the objectives of science communication, generally stating the guiding effort of their communication as informing and educating an audience [35, 47, 51]. Communicating with the objective to inform in order to achieve the greater societal goal of increased science literacy is what has become known as the “deficit model” of communication [52]. However, available evidence does not support the idea that information or lack-of is key to affecting audience attitudes towards science or inspiring changes in decision-making behavior. Recent studies conducted by Besley et al. [31–33] have surveyed scientists located in North America to determine how scientists prioritize different communication choices. These studies have indicated that the belief that a choice would be effective (e.g., tactic [50]; goal [33]) and whether that choice is viewed as ethical (e.g., tactic [32]; objective [31]) positively influences their willingness to prioritize that choice. This line of research then has indicated the likelihood that evidence-based recommendations, and discussions about the ethicality of different choices, would be effective at shifting scientists’ communication choices.

#### Perceptions of scientists

The stereotype content model captures group stereotypes along two dimensions of social cognition–warmth and competence [53, 54]. Both of these dimensions have been shown to be important in effective science communication [55]. While scientists are held in high regard as experts, falling high on the competence dimension, they are viewed as lacking in terms of warmth, and correspondingly, trustworthiness [55]. Along with competence, trust is typically measured as a perception of warmth, which is an amalgamation of traits such as openness, honesty, sincerity, and sociability [53, 56]. However, instead of automatically being seen as trustworthy, scientists face stereotyped perceptions of being aloof, cold, and “valuing knowledge over morality” [12, 13]. This poses a communication challenge for scientists as audiences typically judge the warmth of a communicator before judging their competence when choosing whether to pay attention or believe the information being communicated to them [9]. Survey research has found trust in scientists to be an important factor in shifting public attitudes across a broad range of topics, including nanotechnology [57] and climate change [58].

Scientists therefore need to counter these stereotypical perceptions of who people in their profession are. They can do this through a process of individualization, depicting themselves as good-intentioned individuals that share beliefs and experiences with others [16]. Therefore, scientists would benefit from using tactics that allow for two-way engagement with the public and show their individual personalities to target the objectives of increasing their perceived warmth and trustworthiness while not harming their perceived expertise or competence.

#### Video and online science communication

A science-curious public can learn about news from a variety of different places, from traditional sources such as newspapers, television, and online news sources, to newer mass media in the form of blogs and social media platforms [1]. Traditional science journalism has been decreasing in recent decades, becoming increasingly overtaken by online media outlets [2, 3]. Blogs, webpages, and social media however have seen a surge and the science stories presented there can be told by many individuals, from interested non-scientists, spokespeople with science backgrounds talking about the work of others, to the scientists themselves (e.g., [3–5, 7, 25]). The shift from traditional to non-traditional sources for science news also comes with a shift from a one-way dialogue towards two-way engagement providing greater access to the content producers themselves.

Online videos are increasingly being recognized as an effective and popular medium of science communication for both professional and non-professional content producers [24–26]. In fact, recent experimental work found that video was more effective than traditional written media at conveying the concept of scientific consensus on global climate change (video vs. written communication [59]) and positively impacted audience comprehension, perceived pleasantness, and expressed interest in response to human disease-related research stories (video vs. written press-release [60]). Additionally, “optimized video” that was designed with key features (e.g., narrative structure, non-technical language) for optimal comprehension and engagement was more effective in all measures than “non-optimized” video [60]. Such “optimized” videos are often the most popular on online video platforms, such as YouTube.

With 2 billion monthly users, and over a billion hours of video watch daily, YouTube is a leading platform for video content [22]. Several studies have analyzed science-themed YouTube videos to better understand content and engagement characteristics of videos that perform well on the platform. Popular videos have been found to focus on storytelling, have a moderate amount of production value, and emphasize personality and a direct connection with an audience [27]. Professional producers of popular YouTube science channels highlight the platform as a unique space for a direct connection and community between viewers and producers [61]. In fact, audience engagement indicators such as likes and comments have been shown to correlate with popularity of science content [6]. These and other analyses of YouTube content often focus on the most popular videos and channels which are overwhelmingly produced by professional content creators, not by scientists who are self-sharing their work (e.g., [5]). In fact, there are few active researchers who maintain presences on YouTube, which likely stems from the time investment it demands and a correspondingly perceived lack of institutional and collegial support [39]. Additionally, as scientists’ self-efficacy assessments correspond to their efforts to publicly communicate and the tactics and objectives they prioritize [31, 32, 39], it is likely that negative self-efficacy assessments by scientists are contributing to hesitancy to create and post public-oriented videos of their research. However, the few studies that have considered scientist-presented online video point towards scientists being especially effective.

Online videos of TED talks (the Technology, Entertainment, and Design conference) presented by academics have been found to garner more engagement with general audiences than those by non-academics [62]. On YouTube, TED videos with academic researchers presenting received more comments and more likes than those that featured presentations by non-academics [63]. Two recent experimental studies are, to our knowledge, the only to compare how scientists-as-presenters in online video compare in effectiveness to non-scientists. Reif et al. [29] showed 1-minute-long clips of four television-produced interviews with scientists and two clips of professional science YouTubers to survey respondents. Perceptions of integrity and benevolence did not differ across the stimuli, however, YouTube science presenters, as compared to scientists, were viewed as less competent but more entertaining and comprehensible when talking about physics. Finally, Davis et al. [28] surveyed responses to climate change themed video narrated either as ‘infotainment’ or an expository style. In the infotainment style, the narrator, self-identified as a scientist, presented the information in the form of a personal humorous story. The researchers modeled the infotainment treatment after the style of popular user-generated videos on YouTube. The expository narration was delivered in a more traditional documentary style, in an unidentified third-person voice with formal language and a serious tone. Respondents indicated liking and believing the expository treatment significantly more than the infotainment. Whereas the infotainment delivery was more liked by viewers without a college education and made viewers better equipped for correctly answering three of four information recall questions. In both experiments, confounding variables between treatments were not controlled for, making an assessment of the tactic of scientist-as-presenter difficult.

### The current study

Here, we explore how the tactic of putting a person on-screen and varying their relationship to the work they are presenting (i.e., varying the narrative perspective) affects both short-term objectives and long-term goals that are vital to effective science communication. Specifically, our communication goals relating to improving warmth, and correspondingly trustworthiness, perceptions of scientists in general and increasing support for basic science research. To do so, we crafted video narratives that represent common ways in which public audiences first encounter new research science, to follow the recommendation from the National Academies of Sciences, Engineering, and Medicine [9] to mimic real-world communication scenarios. Therefore, these video narratives are told from either the perspective of a scientist presenting their own work, a third-party spokesperson summarizing research results, or an infographic-type video using third-person text on-screen without an on-screen presenter and no audio narration. All these narratives told the same fictionalized science story about the discovery of a new ant species using museum specimens. We expected that due to the individuation process, the tactic of having scientists presenting their own work, would improve the trustworthiness of the spokesperson, enjoyment of the video content, humanize research, and in turn foster improved warmth perceptions of scientists in general, and attitudes towards research and funding over viewing a third-party spokesperson or a video with no on-screen presenter. Therefore, we have the following research questions and hypotheses split between short-term objectives and long-term goals.

Objective research questions and hypotheses:

RQ1,2: How does viewing a video in which the scientist presents their own research material influence audience perception of the trustworthiness of the spokesperson and video content?
H1: Scientists presenting their own work will be perceived as having more expertise than third-party spokespersons’ presenting the work of others.
H2: Viewing videos in which a person is presenting the science story will be rated more enjoyable than when there is no spokesperson on-screen to tell the science story, with the most enjoyable treatment being when the scientist presents their own work.
H3: Scientists presenting their own work will lead to more respondents describing the informational content of the video with terms that also include the researchers (e.g., “she/he discovered that…”, rather than “a new ant was discovered”).

Goal hypotheses:

H4: Viewing a scientist presenting their own research will positively influence perceptions of the warmth of scientists in general compared to when a third-party spokesperson presents the information and when there is no spokesperson on-screen.
H5: Stimulus enjoyment will positively influence attitudes toward and funding for basic science research and museum natural history collections.

Additionally, we explore whether there is a difference in perception of the competence of scientists in general across treatments although we do not expect there to be a difference as scientists are rarely viewed as being incompetent.

## Materials and methods

This study was approved by the North Carolina State University Institutional Review Board (IRB# 20994). In June of 2020, participants were recruited via Qualtrics, an online survey hosting platform that uses volunteer research participants and compensates participants who submit survey responses. The only requirement to participate in the survey was being able to give informed consent (i.e., at least 18 years of age).

**Fig 1:**
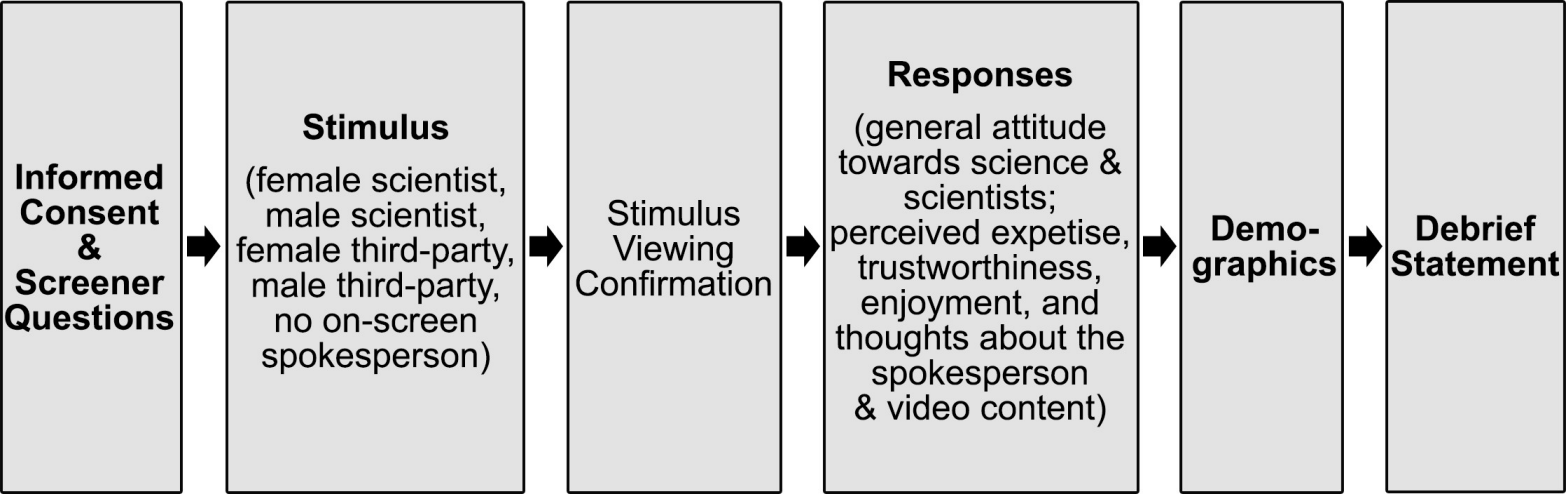
Experimental design. Participants were randomly assigned to one of the five video stimuli in equal proportions.

Once informed consent was given, participants were randomly assigned one of the five stimuli, and asked to read the accompanying text and watch the entire video (Fig 1). After viewing the stimulus, participants answered attention check items to ensure they viewed the stimulus before proceeding with answering questions regarding their general attitude towards science, and their views about the video, spokesperson, and video content. Finally, participants finished the survey by answering some demographic questions and then viewing a debrief statement.

### Sample

The final sample consisted of 515 people ranging in age from 18 to 87 (mean = 47). Most respondents were not of Hispanic or Latino descent or origin (90.3%) and identified as white (81.9%). Most respondents also identified as female (64.5%) with two participants not identifying as either male or female. Almost half of the respondents belonged to the Democratic party (47.8%) and considered themselves liberal (49.1% “lean liberal”, “liberal”, or “very liberal”). About half of respondents had a college degree or higher (49.7%) though most of their degrees were not in STEM fields (88.0%). Overall, more participants had gross annual household incomes under $55,000 a year (55.9%) compared to over $55,000 a year (44.1%). For more detailed demographic breakdown of respondents see S1 Appendix.

### Stimuli

We created five video stimuli that fall into three treatments: scientist presenting (first-person), third-party spokesperson (third-person), and no on-screen spokesperson (third-person) (Table 1; see S1 Appendix for scripts and screenshots of stimuli). Two videos were created for each treatment that included a person on-screen (one male-presenting, one-female presenting) to help control for idiosyncratic effects of the individual presenter. Presenters differed in age (male = 36, female = 29) and whether they were of Hispanic or Latino dissent (male = no, female = yes). Each presenter recorded a video as a scientist and a third-party, appearing on-screen talking directly to the audience between 25–29% of the total runtime of the video. The only differences between our first- and third-person stimuli were 14 instances in the script where the person on-screen either makes an “I” or “my” statement versus saying “scientists” or “researchers”, and a “Dr.” title with their fictionalized gender-neutral name, Jaimie Miller. The no spokesperson on-screen treatment followed the third-party treatment script and neither one of our presenters appeared on-camera or via recorded audio.

**Table 1:** Summary of video stimuli, treatment, video length, and number of complete responses.

Video stimuli	Treatment (language)	Video length	Number of responses
Male scientist	Scientist (first-person)	2:27	102
Female scientist	Scientist (first-person)	2:24	104
Male third-party spokesperson	third-party spokesperson (third-person)	2:32	104
Female third-party spokesperson	third-party spokesperson (third-person)	2:28	103
No on-screen spokesperson	No on-screen spokesperson (third-person text)	2:22	102

Each of these videos presented the same science story and were presented with a three-sentence blurb. The first two sentences were the same across all treatments: “What was previously thought of as one ant species is now two. By looking in museum collections, and focusing on understudied male ants, researchers have discovered a new species of trap-jaw ant.” The third sentence depended on the treatment. For the first-person scientist treatment it read, “The researcher who made this discovery explains their findings in this video.” For the third-party spokesperson and no on-screen spokesperson treatments it read, “The discovery is explained in this video.”

Videos were filmed on the same day, in the video production studio located at the North Carolina Museum of Natural Sciences, with b-roll added from existing footage filmed by Adrian Smith, or shot in the North Carolina State University Insect Museum. Footage and audio were edited using Adobe^Ⓡ^ Premiere Pro (version 14.0) and Audacity^Ⓡ^ (version 2.3.3). Videos were all approximately two and a half minutes long (range 2:22–2:32; Table 1).

A within-subjects manipulation check was conducted prior to the experiment to ensure the stimuli accurately reflected the desired manipulations (see S1 Appendix). Open-ended feedback was also collected to make any necessary modifications to the videos. Twenty-three participants were recruited through university affiliated listservs. Results indicated strong manipulations with 22–23 participants for each video accurately identifying whether a person presented the information in the video and whether that person identified as the scientist who conducted the research. Based on the open-ended feedback, music was added to all video stimuli to better reflect “real life” audience expectations of these types of videos.

### Science story

The science story told in our video stimuli was a fictionalized research study crafted to mimic a research news story presenting both the findings and their implications. This story described the discovery of a new ant species highlighting the importance of maintaining and preserving museum collections which are generally publicly funded. This narrative was based on two real ant species and the real differences between them (*Odontomachus clarus* and *Odontomachus relictus* in [64–66]), but aspects of the story, such as how the new species was discovered and who made that discovery, were fabricated. The topic was chosen for practical reasons: (a) control over crafting the scientific narrative, (b) ability to film b-roll material to include in the visual stimuli, and (c) as a somewhat neutral, if not esoteric, topic that audiences are not likely to have strong prior beliefs towards. Ants for example, are not liked but neither are they most hated insect [67]. Choice of a different insect taxon such as butterflies which are viewed as beautiful [68] or bees which are recognized as important pollinators (e.g. [68, 69]) or a charismatic mammal, may have evoked stronger positive emotions from participants which could have impacted audience perceptions (e.g. through emotionalization, mechanisms reviewed in [70]). Overall, the communication goal of this topic was to increase support for museum collections and basic natural history work. Museums themselves tend to be undervalued [71] and underfunded [72, 73] despite being acknowledged as important (e.g., [74]) and having an increasing role in many research fields [71, 75, 76]. Additionally, basic natural history is generally undervalued, such as the taxonomy work needed to describe new species [73].

### Measures

We assessed participants’ attitudes towards science and deference to scientific authority as well as perceptions of scientists in general, the stimulus, and the spokesperson using existing scales, summarized in Table 2. We also assessed participants’ attitudes towards the research and funding of basic science research and museum collections. Additionally, we assessed whether the stimulus viewed humanized the research (Table 2; see S1 Appendix for survey wording and full question list).

**Table 2:** Summary of the scales, how they were measured, their sources, and Cronbach’s alpha.

Scale	How measured	Source of scale	Cronbach’s alpha (M ± SD; scale items)
Overall attitude towards science	Rate statements on a 7-point scale (strongly disagree to strongly agree)	National Science Board [77]	0.82 (5.78 ± 0.98; 4-item: “Even if it brings no immediate benefits, scientific research that advances the frontiers of knowledge is necessary and should be supported by the federal government.”; “Because of science and technology, there will be more opportunities for the next generation.”; “Scientific research can help to address many of our environmental issues such as air and water pollution.”; “Scientific research can help to address many of our health issues such as cancer and access to affordable health care.”)
Deference to scientific authority	Rate statements on a 7-point scale (strongly disagree to strongly agree)	Brossard & Nisbet [78]	0.79 (5.08 ± 1.10; 4-item:”Scientists know best what is good for the public.”; “It is important for scientists to get research done even if they displease people by doing it.”; “Scientists should do what they think is best, even if they have to persuade people that it is right.”; “Scientists should make the decisions about the type of scientific research on conservation.”)
Spokesperson trustworthiness	Evaluate five opposite word pairs on a 7-point scale	Word pairs from Miller et al. [79] which was adapted from McCroskey et al. [80]	0.84 (5.65 ± 1.10; 5-item: dishonest-honest, bad-good, worthless-valuable, selfish-unselfish, sinful-virtuous)
Stimulus trustworthiness	Evaluate six opposite word pairs on a 7-point scale	Word pairs from Kim and Cameron [81] based on Ohanian [82]	0.94 (5.88 ± 1.18; 4-item: accurate-inaccurate, believable-unbelievable, convincing-unconvincing, trustworthy-untrustworthy)
Spokesperson expertise	Evaluate three opposite word pairs on a 7-point scale	Word pairs from Miller et al. [79] which was adapted from McCroskey et al. [80]	0.93 (5.81 ± 1.25; 3-item: inexpert-expert, unintelligent-intelligent, unqualified-qualified)
Enjoyment of stimulus	Evaluate seven statements on a 7-point scale (strongly disagree to strongly agree)	Subset of the intrinsic motivation inventory by Ryan [83]	0.96 (4.47 ± 1.57; 5-item: “I enjoyed this video very much”, “This video was fun to watch”, “I would describe this video as very interesting”, “I thought this video was quite enjoyable”, “While I was watching this video, I was thinking about how much I enjoyed it”)
Humanizing research	Asked to describe what they saw and heard following viewing the stimulus. Responses were coded as whether a person or people were referred to in recalling the content of the stimulus (1 = yes, 0 = no)	This paper	—
Warmth of scientists	How well 12 words describe traits of scientists in general on a 5-point scale (not at all to extremely)	Reported in Jarreau et al. [15] that was derived from Fiske’s work on scientist stereotypes [55]	0.90 (3.62 ± 0.68; 9-item: sincere, honesty, warm, helpful, sociable, ethical, likeable, friendly, trustworthy)
Competence of scientists	How well 4 words describe traits of scientists in general on a 5-point scale (not at all to extremely)	Reported in Jarreau et al. [15] that was derived from Fiske’s work on scientist stereotypes [55]	0.65 (4.21 ± 0.64; 3-items: competence, confidence, intelligent)
Attitude towards research	Evaluate three statements on a 7-point scale (strongly disagree to strongly agree)	This paper	0.89 (5.60 ± 1.09; 3-item: “Even if it brings no immediate benefits, scientific research, like this study, is necessary and important.”, “Scientific research, like this, that describes new species is scientifically important.”, “Scientific research, like this, done for the sole purpose of advancing the frontiers of knowledge benefits society.”)
Attitude towards funding	Evaluate three statements on a 7-point scale (strongly disagree to strongly agree)	This paper	0.88 (5.14 ± 1.33; 2-item: “Museum natural history collections, such as featured in this video, should receive public taxpayer support.”, “Scientists who work in natural history collections, such as featured in this video, should receive public taxpayer support.”

scale mean (M) ± standard deviation (SD)

### Statistical analyses

Analyses were conducted in SPSS Statistics (version 26; IBM). Hypotheses were tested using ANCOVA, Pearson correlations, and a chi-square test in SPSS. ANCOVA assumptions of homogeneity of error variances and normality of the residuals were checked using Levene’s tests and visually using Q-Q plots respectively. As we conduct multiple ANCOVA models, we calculated adjusted *P* values (also referred to a *Q* values) to account for false discovery rates [84] using the *p*.*adjust* function in the base *stats* package (method = “fdr”) in R (version 3.6.1 [85]). These *P* values are adjusted across all ANCOVA models reported in Table 3 and Table C in S1 Appendix.

**Table 3:** ANCOVA and post-hoc analyses of the effects of treatment (scientist vs third-party vs no on-screen spokesperson) on outcome measures.

	Estimated marginal mean (standard error)					
	Scientist	third-party	No on-screen spokesperson	F(d1,d2)	*P* value (adjusted *P* value)	Partial eta squared
**Spokesperson**						
Expertise	6.04 (0.09)_a_	5.67 (0.09)_b_	5.63 (0.12)_b_	6.13 (2, 510)	0.002 (0.03)	0.023
Trustworthiness	5.81 (0.08)_a_	5.61 (0.08)_ab_	5.40 (0.11)_b_	5.07 (2, 510)	0.007 (0.05)	0.019
**Stimulus**						
Trustworthiness	5.96 (0.08)	5.82 (0.08)	5.84 (0.12)	0.81 (2, 510)	0.45 (0.89)	0.003
Enjoyment	4.47 (0.11)	4.45 (0.11)	4.53 (0.16)	0.10 (2, 510)	0.90 (0.94)	0.000
**Scientists**						
Competence	4.23 (0.04)	4.21 (0.05)	4.21 (0.06)	0.07 (2, 510)	0.94 (0.94)	0.000
Warmth	3.64 (0.05)	3.64 (0.05)	3.52 (0.07)	1.24 (2, 510)	0.29 (0.68)	0.005
**Attitudes**						
Research	5.65 (0.07)	5.56 (0.08)	5.60 (0.11)	0.37 (2, 510)	0.69 (0.94)	0.001
Funding	5.17 (0.09)	5.16 (0.09)	5.02 (0.13)	0.48 (2, 510)	0.62 (0.94)	0.002

Adjusted *P* values account for false discovery rates.

Lowercase subscript letters denote significant post-hoc pairwise comparisons with Bonferroni adjustments at or below the *P* < 0.05.

Visualizations were conducted in R using the following packages: haven (version 2.2.0 [86]), tidyverse (version 1.3.0 [87]), and ggplot2 (version 3.2.1 [88]).

## Results

We investigated the effects of treatment (scientist, third-party, no on-screen spokesperson) on perceived spokesperson expertise, trustworthiness of the spokesperson and stimulus, stimulus enjoyment, warmth and competence of scientists in general, and attitude towards natural history research and museum collection funding (S1 Dataset). We tested each of these individually with ANCOVA analyses, with demographic variables included as covariates. To determine which demographic variables should be included as covariates, we conducted ANOVA and chi-square tests on individual demographic variables to determine which variables differed across treatments (see S1 Appendix). Only age (continuous) and ideology (categorical binary: conservative vs. liberal) were significantly different across treatments. The Pearson correlation between age and ideology covariates was checked prior to analyses. Age and ideology exhibited a significant but weak correlation (r = -0.090, *P* = 0.042), thus both were included as covariates in ANCOVA models with both *P* values and adjusted *P* values reported. Post-hoc comparisons were conducted on significant fixed effects with Bonferroni adjustments.

### Influence of presentation treatment on communication objectives

**Fig 2:**
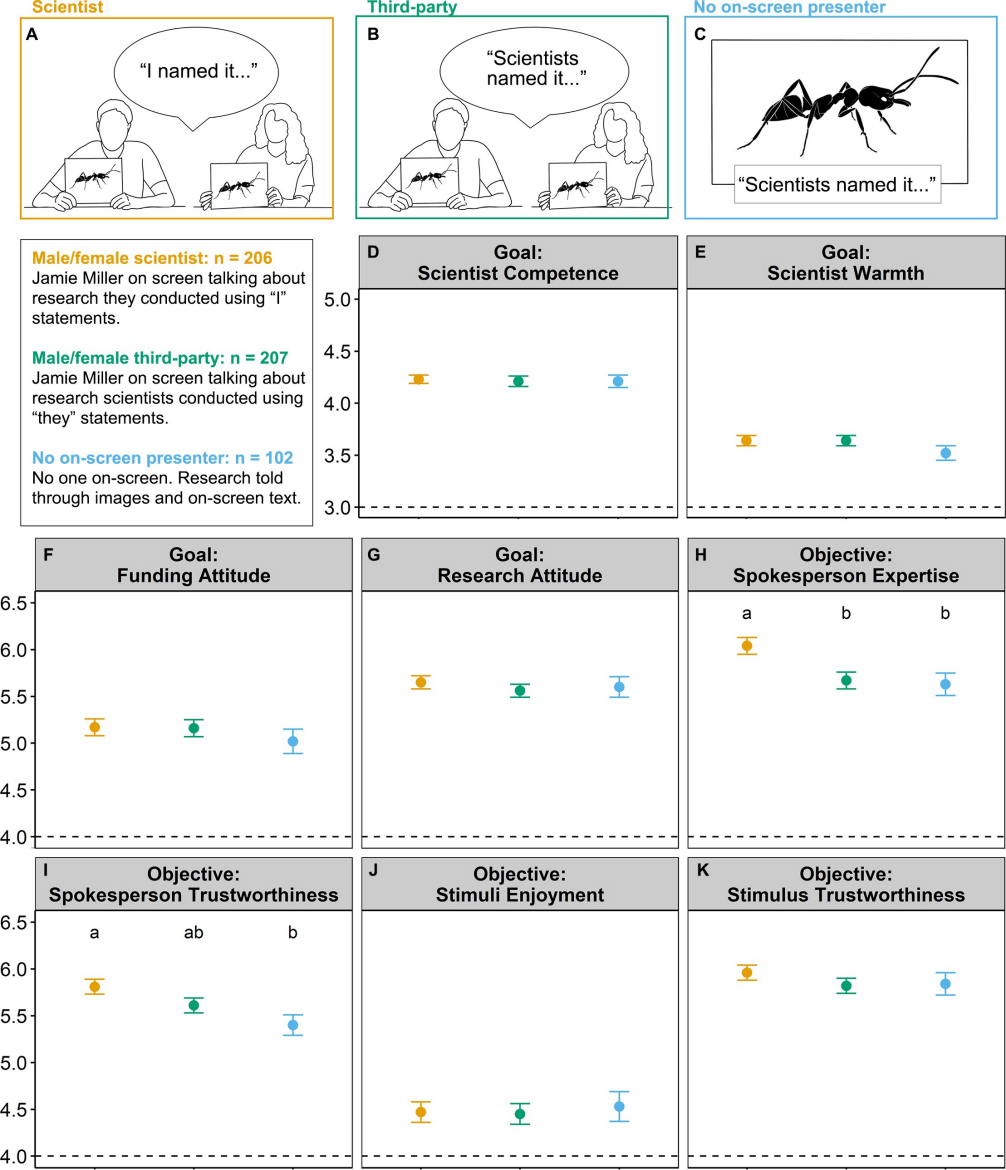
Experimental methods and marginal mean ± Standard Error (SE) of outcome measures across treatment from ANCOVA analyses. Experimental treatments (A-C) and their corresponding results (matching in outlined color in panels A-C). Perceptions of the competence (D) and warmth (E) of scientists in general were rated on a 1 to 5 scale with values closer to 5 indicating traits that represent scientists in general. Attitudes towards funding (F) and research (G), as well as perceived spokesperson expertise (H), spokesperson trustworthiness (I), stimulus enjoyment (J), and stimulus trustworthiness (K) were rated on a 1 to 7 scale with values closer to 7 expressing more positive perceptions. Dotted line on all graphs indicates the neutral response scale midpoint. Letters indicate significant differences based on post-hoc tests with Bonferroni adjustments significant at the *P* < 0.05 level.

In answering RQ1 and RQ2, we found treatment did have a significant effect on perceived spokesperson trustworthiness (*F*_2,510_ = 5.07, unadjusted *P* = 0.007, adjusted *P* = 0.05; Fig 2I, Table 3). Participants rated the scientist treatment the highest on spokesperson trustworthiness with the no on-screen spokesperson treatment the lowest and third-party spokesperson in the middle (scientist vs. third-party: *P* = 0.20; scientist vs. no on-screen spokesperson: *P* = 0.006; third-party vs. no on-screen spokesperson: *P* = 0.33). Stimulus trustworthiness did not differ across treatment (Fig 2K, Table 3).

Treatment did have a significant effect on perceived spokesperson expertise (*F*_2,510_ = 6.13, unadjusted *P* = 0.002, adjusted *P* = 0.03; Fig 2H, Table 3). Participants rated the scientist treatment the highest on spokesperson expertise with both the third-party (scientist vs. third-party: *P* = 0.007) and the no on-screen spokesperson treatments lower (scientist vs. no on-screen spokesperson: *P* = 0.017; third-party vs. no on-screen spokesperson: *P* = 1.00). Thus, H1 was supported.

Treatment did not have a significant effect on stimulus enjoyment (Fig 2J; Table 3). H2 was not supported.

There was a significant effect relationship between treatment on whether participants described the research in terms that included the researchers (χ^2^_2_ = 8.41, *P* = 0.015). Thus, H3 was supported. Respondents mentioned a person or people in the descriptions of stimuli they viewed 42.75% (86/206), 33.33% (69/207), and 34.21% (26/102) of the time when they viewed the scientist, third-party, and no spokesperson treatments respectively (Table 4). Some examples of what participants said when mentioning that scientists or researchers took part in the research included: “This researcher has di[s]covered an additional species of rare ant in Florida”, “How scientists discovered what they thought was one species of trapjaw ants is actually two different species”, “They discovered a new species of ant hiding in plain sight”. Participants that did not mention a person and people in their response focused more on the content of the video, for example: “A new ant discovery and why museums are needed for research needed”, “Discovery of a new species of ant by accident by looking at male ants.”, “A new species of ant was discovered based on comparison, due to previous collections with a museum”.

**Table 4:** Humanizing research results. Observed counts of respondents recalling a person or people when describing the stimuli they viewed by treatment.

		Respondents recalled a person or people when describing stimuli		Total
		No	Yes	
**Treatment**	**Scientist**	120	86	206
	**Third-party**	138	69	207
	**No on-screen spokesperson**	76	26	102
**Total**		334	181	515

### Influence of presentation treatment on communication goals

Treatment did not have a significant effect on the perception of scientists in general as warm or competent (Fig 2D and 2E; Table 3). These findings do not support H4 but are consistent with our expectations that scientists are generally perceived as competent.

Treatment did not have a significant effect on attitudes towards natural history and museum collections research or funding (Fig 2F and 2G; Table 3). However, there were moderate and significant positive correlations between stimulus enjoyment and attitude towards research (Pearson correlation: r = 0.47, *P* = 0.00), and stimulus enjoyment and attitude towards funding (Pearson correlation: r = 0.42, *P* = 0.00). Thus, H5 was supported.

## Discussion

This study is the first of our knowledge to experimentally control and test the impact of scientists acting as presenters in online video media on public perceptions of science. Specifically, we sought to assess how scientists can use the strategic communication tactic of putting themselves on-screen talking about their own work with the objective of improving public perceptions of scientist communicators with the long-term goals of combating negative scientist warmth stereotypes and raising support for, and funding of, natural history and museum collections. We found that scientists presenting their own work on-screen can positively influence short-term objectives related to spokesperson trust and expertise, as compared to when the same science being presented through other means. However, these differences in audience attitudes did not correspond to our communication goals of more positive feelings towards science and scientists in general.

Typically, participants are reluctant to rate individuals negatively, therefore the more individuated the person, the less likely they are to receive negative views [55, 89]. This could explain why participants rated our scientist spokesperson as highest on trustworthiness and expertise scales compared to the other treatments. Our scientist treatment where the scientist appeared on-screen and gave their own direct account of the information using “I” statements, was the most individuated treatment that participants could have been exposed to. The no on-screen spokesperson was our least individuated treatment, as information was conveyed in an infographic-like manor with text and images on-screen and no voice over. Our third-party spokesperson treatment falls in the middle, having an individual on-screen summarizing the results and implications of unnamed scientists’ research. Additionally, participants may have been more hesitant then to rate our scientist spokesperson negatively compared to when they were evaluating scientists as a group.

Context may also play a role in how specific scientists versus scientists in general are viewed. For example, Fiske & Dupree [55] demonstrate that scientists and researchers who are not viewed as public-communicators fall lower on the warmth, and consequently trustworthiness, scale than scientists who also do a form of public-communication (e.g., professors and teachers). Therefore, our scientist spokesperson may be rated differently, and perceived categorically differently, then a generalized scientist. Alternatively, Besley et al. [56] noted that support for specific research fields (e.g., genetically modified research) was different depending on whether participants evaluated scientists within the general context of “research at American universities” or specific context of “research at American universities on genetic modification of food crops” [56]. For example, benevolence, which is a component of trustworthiness, was not important in a general context but was in a specific context, suggesting that perceptions of scientists in general does not necessarily indicate that the public will hold the same perceptions of scientists conducting specific research. It is possible that our basic natural history research focus is evaluated differently than both research in general or more applied research topics.

Our long-term goals may not have been impacted by our treatments because participants only took part in a single experiment in which they viewed a single stimulus. The tactics we employed (varying the narrative perspective to encourage different levels of individualization) were better equipped to influence important objectives for effective science communication instead of communication goals. To influence communication goals, it would likely be better to have participants have repeated exposures to scientists presenting themselves as individuals to counter stereotyped perceptions. It may therefore make sense that the warmth of scientists in general were impacted in Jarreau et al. [15]’s paper as participants viewed selfies from multiple different scientists and thus individuated multiple scientists, perceiving each individual as warmer, and then using that entire group to reassess their perception of scientists in general.

### Implications

Our findings present several theoretical and practical implications. Foremost, it adds to literature on narrative perspective and lends support to existing research that found that first-person narratives positively influence audience perceptions of the speaker [23]. A potential mechanism for this effect is narrative engagement–meaning it is possible audiences are more engaged in the story if the narrator is directly involved in it.

This study also bridges scholarship on narrative perspective and public perceptions of scientists, suggesting that having scientists share their own stories and discoveries through online video may be one avenue of mitigating negative perceptions of scientists’ warmth or other personal characteristics. This may be particularly true in instances where a scientist spokesperson defies preexisting beliefs or stereotypes about scientists in general. These findings lend support for expectancy violations theory, which posits that individuals have expectations for communication experiences and their perceptions of the source are relative to those expectations [90]. If the expectancy violation is a positive one, it will result in positive perceptions.

This study may also have implications for the application of the ELM to digital science communication. Depending on the topic being communicated, audiences are more or less likely to elaborate on the message. Our findings support the notion that a scientist’s relationship to the research may act a heuristic in peripheral route processing because the low personal relevance to the audience. However, this may vary depending on topic and individual differences among audience members. More research is needed in this regard.

From a practical standpoint, our results suggest that scientists may receive a trustworthiness boost by putting themselves on-screen to talk about their own work. While few scientists currently run their own YouTube channels, many scientists are already making videos of their work. These videos, however, are primarily intended for peer-scientist audiences instead of for a science-curious public. For example, an increasing number of scientists are creating video summaries (video abstracts) that accompany the publication of their peer-reviewed articles [91]. These videos, while typically posted in public platforms such as YouTube, are embedded in journal websites where they are primarily watched by professional audiences. Correspondingly, when the effectiveness of these video are evaluated, it has been through assessing correlations with increased downloads of the primary paper or higher number of paper citations instead of metrics that would be associated with non-scientist viewership [92]. In addition, the *Journal of Visual Experiments* (*JoVE*) publishes experimental methods in video format, providing unique views into the scientific process [93]. However, again, the intended primary audience for this content is professional peers, not a science-curious public.

Scientists could merge producing their own videos where they appear on-screen with other forms of social media that they may be more familiar with. For example, many scientists and scientific societies use Twitter [17, 94, 95], Facebook [94, 96], and Instagram [94], to communicate both with other scientists and the public [94]. These other social media platforms could be used to advertise and share either newly created channels or videos for further promotion (e.g. [7, 97]) much like how they are currently used to promote blogs (e.g. [98]) or publications ([18, 96, 99]). However, some social media outlets are easier for sharing links than others. For example, Twitter and Facebook are easier to hyperlink to other sources than Instagram [20].

Despite the potential trustworthiness benefit of portraying oneself on-screen, there is a caveat that not all spokespeople are treated equally on the internet. This is important to address when recommending that scientists should communicate their own work. For example, open and anonymous comment sections have led to female science content producers receiving a higher proportion of hostile and sexist comments [100].

### Limitations and future research

As with all research, this study has limitations and presents opportunities for continuing research. We used an opt-in volunteer-based survey panel, which was appropriate because the purpose of our study was to test the effects of experimental treatment. However, future research in this area would benefit from a probability sample in order to make population-based inferences.

Another limitation stemmed from the stimulus design. The finding that first-person narratives were perceived as more trustworthy than third-person narratives could also be attributed to the spokesperson being identified through on-screen, in-video text as “Dr.” in the first-person factor. Future research should control for title or salutation differences or introduce an additional treatment.

More research must also be done on the myriad variables that may influence the communication effectiveness of an individual scientist. We used two different individuals (one male presenting, one female presenting; see S1 Appendix) for the videos to help mitigate idiosyncratic effects of an individual presenter but two scientists are not representative of the population of scientists. Additionally, gender presentation could not be compared because of other potential confounding variables (e.g., spokesperson age, whether of Hispanic descent, appearance, and performance). Future research should test how these individual differences may interact with narrative perspective as spokesperson gender itself has already been demonstrated to influence source credibility (e.g., [44]) and mitigate negative warmth stereotypes of scientists (e.g., [15]).

Additionally, we assessed a single narrative script accompanied by all the same ant visual media. While this allowed for control confounding variables across treatments, it is possible that other narration forms or use of alternative visual cues would have impacted audiences differently potentially though emotionalization. Emotionalization can influence audience attitudes through many different mechanisms regardless of whether the audience is aware or not of their emotional state, or if the emotion evoked is relevant or not to the content of the communication (reviewed in [70]). With the increase in emotionally charged media and communications [101], studies have focused on how reports are written often varying how content is presented either in an emotional narrative form or rational fact-based form (e.g. [102, 103]). However, as visuals can also evoke emotions, use of visual cues can interact with the text to impact outcomes (e.g. knowledge gain in [102]). Therefore, future research should test if different visual cues embedded within different narrative perspectives interacts with audience perceptions.

Finally, the narrative we created centered around the communication goal of building support towards research science and did not focus on other potential communication goals that seek to alter the behavioral of participants. In other words, we did not ask participants to change their beliefs around controversial and personally relevant topics or to make changes in their every-day decision making. There is an existing body of literature the demonstrates that information is processed differently depending on the degree of personal relevance or involvement (e.g., [104–107]). This body of literature recognizes that persuasive outcomes can be influenced both by the degree of personal relevance and by other variables such as source characteristics [104, 105], message argument clarity [104] and strength [105], repetition [107], and emotionalization [108]. Therefore, future research should test whether there is an interaction between narrative perspective, topic relevancy or interest, and communication goal on trust in the spokesperson and in scientists in general.

## Conclusion

Our research has demonstrated that putting a person who identifies as the scientist conducting the research on-screen positively impacts the short-term objectives of increasing trust and expertise which are important for effective science communication. While these have yet to influence long-term goals of shifting perceptions of scientists as a group, it is possible that through increased exposure to individualizations of scientists that over time then perceptions of these long-term goals would change. Similarly, this was only one example of the type of natural history research that may be publicly funded. If audiences are repeatedly exposed to trustworthy scientists narrating their natural history research and discussing how museums contributed, the long-term goal of continued funding for these resources may be impacted. These findings therefore help provide experimental evidence on the impact of prioritizing communication objectives other than informing, highlighting the importance of science communication training for scientists.

## Supporting information

S1 Dataset Full dataset for Qualtrics survey. Includes the raw data, a filter variable to remove straight-lining responses, calculated scales, and saved standardized residuals from the ANCOVA models. In this file, “text_only” refers to the “no on-screen presenter” treatment.
(SAV)

S1 Appendix Supporting information for “testing how different narrative perspectives achieve communication objectives and goals in online natural science videos”. Includes production scripts, questionnaire, additional analyses, and Tables A—G.
(DOCX)
