# Supplementary material for: Testing how different narrative perspectives achieve communication objectives and goals in online natural science videos
Source: PLoS One. 2021 Oct 13;16(10):e0257866. doi: 10.1371/journal.pone.0257866 (PMC8513868; doi:10.1371/journal.pone.0257866)
Supplement: S1 Appendix — Includes production scripts, questionnaire, additional analyses, and Tables A—G. (DOCX) [file pone.0257866.s002.docx]

S1 Appendix for

**Testing how different narrative perspectives achieve communication objectives and goals in online natural science videos**

Selina A. Ruzi^1^*, Nicole M. Lee^2^, Adrian A. Smith^3,4^*

Affiliations: ^1^ Department of Applied Ecology, North Carolina State University, Raleigh, North Carolina, United States of America; ^2^ School of Social & Behavioral Sciences, Arizona State University - West Campus, Glendale, Arizona, United States of America; ^3^ North Carolina Museum of Natural Sciences, Research & Collections, Raleigh, North Carolina, United States of America; ^4^ Department of Biological Sciences, North Carolina State University, Raleigh, North Carolina, United States of America

Corresponding Authors*

E-mail: sruzi24@gmail.com (SAR), adrian.smith@naturalsciences.org (AAS)

**This PDF file includes:**

Production scripts

Survey instruments

Supplemental text, tables, figures

Screenshots of video stimuli

References for S1 Appendix citations

**Table of Contents**

**Production Scripts3**

Scientist vs. third-party treatment3

No on-screen spokesperson treatment5

**Manipulation Check7**

Methods7

Manipulation check survey instrument7

Results8

**Qualtrics Panel Survey Instrument10**

**Supplemental Analyses and Results15**

Demographic variables15

Distribution across treatments16

Additional analyses17

General attitude towards science and deference to scientific authority17

Other perceptions of scientists in general17

Influence of spokesperson gender identity on audience perceptions19

**Supplemental References27**

**Production Scripts**

**Scientist vs. third-party treatment**

The scientist treatment is told in first-person, on-screen narration (Fig. 1B,C) while the third-party treatment is told in third-person, on-screen narration (Fig. 1E,F). The changes between the two treatments are highlighted in bold. In both treatments, the person on-screen introduces themselves as Jamie before a title screen appears (Fig. 1A) and all the same supplemental visuals and b-roll was used across treatments (Fig. 1H,I,J,K,L). All treatments had the same ending slide (Fig. 1D).

Hi. I’m Jamie and I want to share with you **my new discovery/ something that scientists have just discovered**, a new species of trap-jaw ant.

These two ants might look the same. And for years **I/scientists** thought that they were. But, this one is actually the newest described species of trap-jaw ant. **I’ve/Scientists have** named it *Odontomachus relictus*.

Trap-jaw ants are famous for their spring-loaded jaws they snap shut to capture prey and defend themselves from predators. In the US, they are found throughout the southern and western states.

For years, **I/scientists** thought this one, found in high elevation sand ridge regions of Florida, was an eastern population of this species, *Odontomachus clarus*, which is common in the southwestern US.

But recently, **I was/researchers were** looking through museum collections and realized **I/they** had been focusing on the wrong things. **My/their** moment of discovery came when **I/they** stopped focusing on workers and started comparing male specimens.

Male trap-jaw ants look nothing like workers and queens which are all female. In nature they are only around during the summer mating season, waiting to fly out of the nest and mate with a new queen. Beyond dispersing and mating, males have little to no role in daily colony life.

Because male ants play minor roles in a colony and are only around at a specific time of year, **I/scientists** usually don’t pay much attention to them. But, in this case, taking a closer look at males revealed a completely different and new species.

Males of this new *Odontomachus relictus* species can easily be identified from all other trap-jaw species in the US by their large eyes, widely spaced ocelli, and unique color pattern. Beyond anatomical differences a genetic analysis revealed species level uniqueness as compared with other *Odontomachus* ants.

For **me/scientists**, the most interesting part of this discovery was realizing how rare these ants are. This new species only lives on a few remnant sand ridges in Florida. That’s why I gave it the species name “relictus”.

**My/Scientists say this** discovery would have been impossible without museum specimens. And this is just one small demonstration of the value of natural history collections. The male samples **I/they** used weren’t originally collected for any specific purpose, beyond recording what was present at a specific place and time. So **I was/they say they were** lucky that other researchers had collected and catalogued enough of these specimens to make this discovery possible.

**My research has/Scientists have** given us a new species of ant and an example of how taxpayer funded museum collections are crucial to our understanding of the natural world.


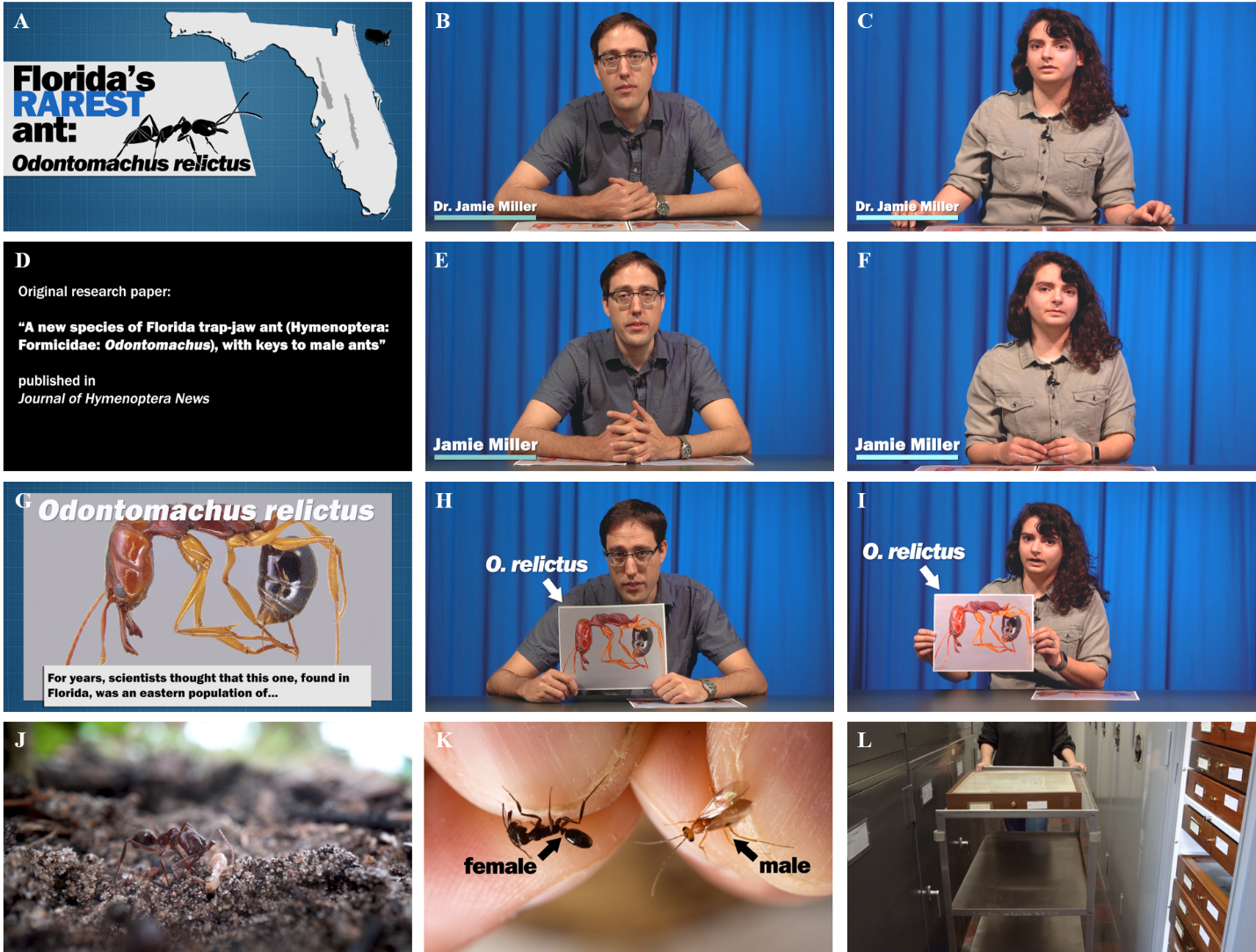


**Fig. 1. Screenshots from video stimuli.** Title slide (**A**) and ending slide (**D**) appearing in all video stimuli. Scientist treatment videos where the on-screen spokesperson used first-person statements to describe their research (**B**, **C**). Third-party treatment videos where the on-screen spokesperson used third-person statements to describe their research (**E**, **F**). The same supplementary visual material was used in treatments that had spokespeople appear on-screen (**H**, **I**) as in the no on-screen spokesperson treatment (**G**). *Odontomachus relictus* photo is modified from MacGown et al. [1]. All individuals featured in the content have given written informed consent. The same b-roll (all filmed by A.A. Smith) were used in all video treatments. Examples of some b-roll include footage of a trap-jaw ant hunting a termite (**J**), a female and male trap-jaw ant (**K**), and a person removing a drawer from a cabinet in an insect collection (**L**).

**No on-screen spokesperson treatment**

The no on-screen spokesperson treatment is presented in third-person written word, with no audio narration. The script appears on-screen as text over supplemental visuals and b-roll video material (Fig. 1G,J,K,L). In general, this script follows that of the language used in the third-party treatment though has slightly shorter sentence length for easier reading. In this treatment, the first thing participants see on-screen is the title slide (Fig. 1A) instead of a self-introduction from a presenter.

These two ants might look the same, and for years scientists thought they were. But, this one is actually the newest species of trap-jaw ant. Researchers have named it: *Odontomachus relictus*.

Trap-jaw ants are famous for their spring-loaded jaws they use to capture prey and defend themselves. In the US, they are found throughout the southern and western states.

For years, scientists thought that this one, found in Florida, was an eastern population of this species, *Odontomachus clarus*, which is common in the southwestern US.

But, looking through museum collections, researchers realized they had been focusing on the wrong things. Their moment of discovery came when they stopped focusing on workers and started comparing male specimens.

Male trap-jaw ants look nothing like workers and queens, which are all female. They are only around during the summer, to fly out of the nest and mate with a new queen. Beyond dispersing and mating, males have little to no role in daily colony life.

Because of this, scientists usually don’t pay much attention to them. But, in this case, looking at males revealed a completely different and new species.

Males of this new species can easily be identified from other trap-jaw species in the US by their large eyes, widely spaced ocelli, and unique color patterns. Beyond anatomy, genetics revealed species-level uniqueness from other ants.

For scientists, the most interesting part of this discovery was realizing how rare these ants are. This ant only lives on remnant sand ridges in Florida. That’s why they gave it the species name “relictus”.

Scientists say this discovery would have been impossible without museum specimens, and that this is one small demonstration of the value of natural history collections. These male ants were first collected just to record what was present at a specific place and time. The scientists were lucky that other researchers had collected these ants, making this discovery possible.

Scientists have given us a new ant and an example of how taxpayer funded museum collections are crucial to our understanding of the natural world.

**Manipulation Check**

**Methods**

In May 2020 we conducted a manipulation check of our video stimuli by recruiting participants via a general email sent to select university-associated listservs. In the recruitment email, we described the project as seeking to better understand how the spokesperson in scientific research videos influences how they are perceived, asking for volunteers to participate in a simple manipulation check of our stimuli. Volunteers viewed a video, answered questions related to the manipulation, and then viewed another video, etc. until all three treatments (female scientist, female third-party spokesperson, and no on-screen spokesperson) were viewed (Fig. 2). The video stimuli were all presented in the same order (female scientist, no on-screen spokesperson, female third-party spokesperson) and each stimuli was presented with their associated three sentence blurb (see Methods – Stimuli subsection in main text). The responses were collected through a Google form that was set to not: (a) collect any direct identifiers, (b) require a Google sign in, or (c) record Google ID. Once the response was submitted, the participant viewed a debrief statement. The only requirement to participate was being old enough to grant informed consent.


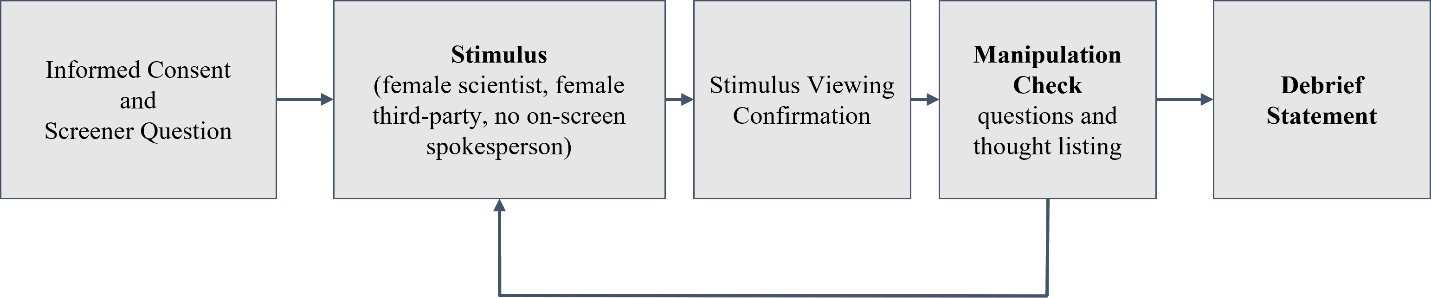


**Fig. 2. Experimental design of the manipulation check.** Every participant viewed all three stimuli videos.

The manipulation check questions asked whether the information in the video was being presented to them by a person and whether it was clear who performed the research. Participants were then asked to take a couple minutes to write any thoughts they had pertaining to the content of the video they watched. These could include their impressions or any other thoughts that might have crossed their minds.

**Manipulation check survey instrument**

Here we present the questions used in the manipulation check survey instrument which was programmed into Google forms.

Informed Consent - on screen and link to a downloadable file

Do you consent to take part in this survey? [Yes, No]

Screener Question: Are you over 18? [Yes, No]

Did you watch and listen to the entire embedded video? [Yes, No]

Is the information in the video being presented to you by a person? [Yes, No]

In this video was it clear who performed this research? [Yes - it was the person in the video, No - the video did not specify who did the research]

Please take a couple minutes to write in the space provided any thoughts you had pertaining to the content in the video you watched. The thoughts you list could deal with your impressions or any other thoughts that might have crossed your mind.

At the end of the survey participants saw the following text:

The study you just completed was designed to examine how the spokesperson in scientific research videos are perceived. While the ant species discussed in the videos you viewed are real species, some of the details regarding their discovery and who made that discovery were fabricated by the researchers specifically for this study and are not factual.

Thank you for your participation and for not discussing the contents of this study with others. If you have any questions about this study, please contact Dr. Adrian Smith as [aasmith7@ncsu.edu](mailto:aasmith7@ncsu.edu).

**Results**

Twenty-three individuals participated in the manipulation check survey. All participants correctly identified that the person who performed the research was in the video for the female scientist stimuli (Fig. 3). All or almost all the participants correctly identified that the video did not specify who performed the research in the no on-screen spokesperson or female third-party spokesperson stimuli respectively. All participants were able to accurately identify that a person presented the information when there was a scientist or third-party spokesperson presenting the information, with only one person saying there was a person presenting the information in the text only stimuli.


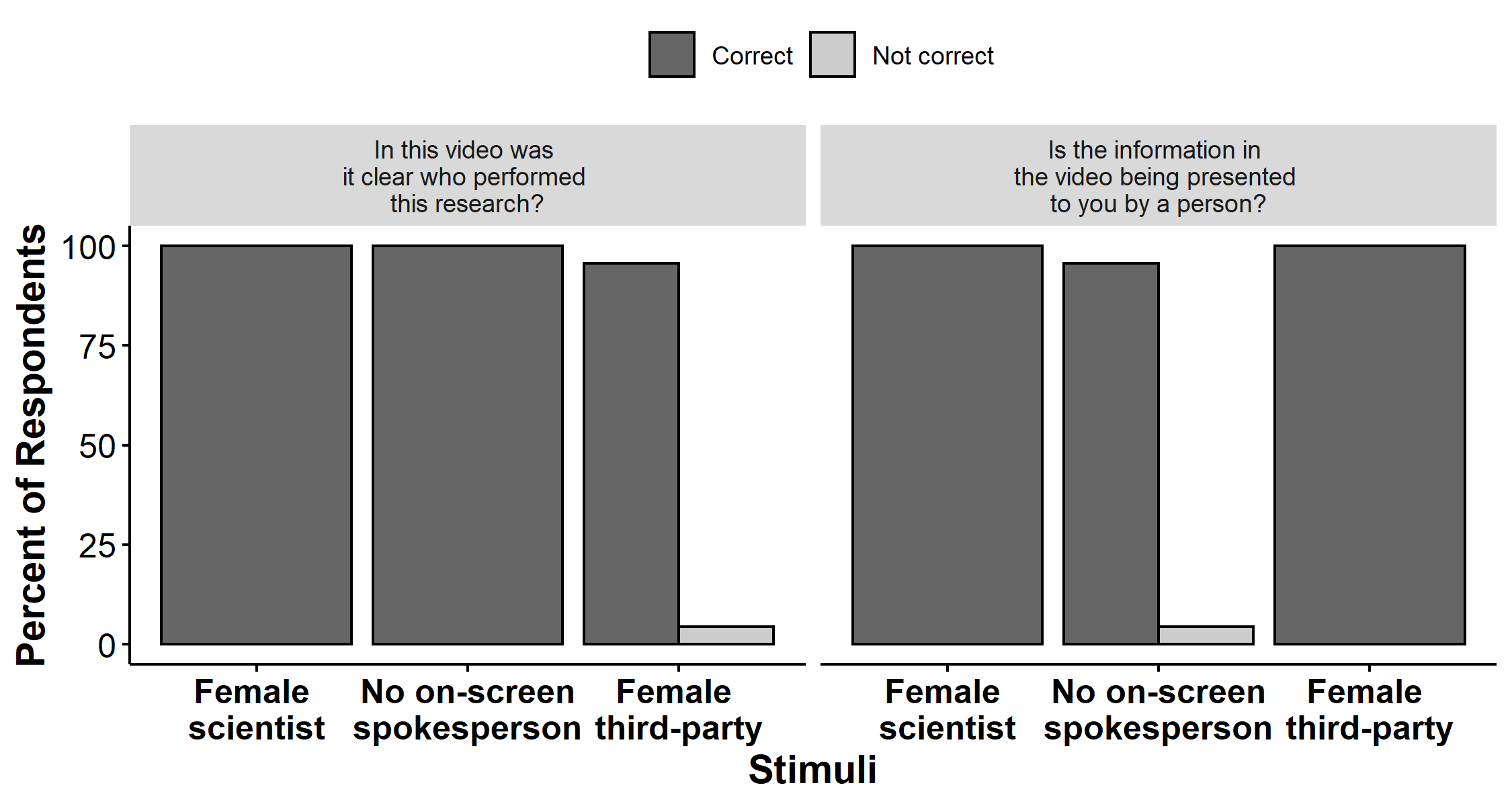


Fig 3. **Manipulation check results.** Summary of whether participants (n = 23) answered correctly if it was clear who performed the research and whether the information in the video was presented by a person.

As part of the survey, participants were asked to list their thoughts after viewing each of the stimuli. For each stimulus, 22 (female scientist and female third-party) or 23 (no on-screen spokesperson) of the respondents provided their comments. Due to the comments about the lack of sounds or background music (most prominent in the no on-screen spokesperson version) we added a background music track (“Gentle Chase” by Podington Bear, licensed from SoundofPicture) to all video stimuli prior to conducting the Qualtrics Panel Survey.

**Qualtrics Panel Survey Instrument**

Here we present the questions used in the instrument and how they were programmed into Qualtrics. All questions required an answer unless otherwise noted and items labeled with an R were reverse coded.

Informed Consent - on screen and as a file download

Screener Question (and included in demographic responses):

How old are you? [dropdown menu]

- Younger than 18 - response terminates survey
- 18:99 - each age as a separate choice
- 99+

Stimuli (one of five videos randomly assigned in equal proportion):

Accompanied with “Please read the following and watch the embedded video.” before the three-sentence blurb and embedded video.

The next arrow to move on to the Stimulus Viewing Confirmation questions does not appear until the length of the video plus 3 seconds of time has elapsed.

Stimulus Viewing Confirmation Questions:

Did you watch and listen to the embedded video? [multiple choice]

- Yes - uses display logic to present the next Stimulus Viewing Confirmation Question
- No - response terminates survey

Tell us briefly but descriptively what you saw and heard (you must enter a meaningful text response based on what you saw and heard to continue the survey). [short answer response, response must have a minimum of 50 characters in order to proceed]

Responses Block

Please state the degree to which you agree or disagree with the following statements. [order of statements presented is randomly decided, 7-point scale from strongly disagree to strongly agree] [Overall attitude toward science from National Science Board [2] statements 1-6; Deference to scientific authority from Brossard & Nisbet [3] statements 8-11]

1. Even if it brings no immediate benefits, scientific research that advances the frontiers of knowledge is necessary and should be supported by the federal government.
2. Because of science and technology, there will be more opportunities for the next generation.
3. We rely too much on science in society today. (R)
4. Scientific research can help to address many of our social issues such as crime and education.
5. Scientific research can help to address many of our environmental issues such as air and water pollution.
6. Scientific research can help to address many of our health issues such as cancer and access to affordable health care.
7. Please select “Agree”. - quality control question, failure to answer correctly terminates survey
8. Scientists know best what is good for the public.
9. It is important for scientists to get research done even if they displease people by doing it.
10. Scientists should do what they think is best, even if they have to persuade people that it is right.
11. Scientists should make the decisions about the type of scientific research on conservation.

Please respond to the statement below by indicating, on a scale of 1 (not at all) to 5 (extremely), how well each of the following words represent traits of scientists. Generally speaking, scientists are… [order of statements presented is randomly decided, 5-point scale from 1 - not at all to 5 - extremely, each term presented as its own statement] [Question reported in Jarreau et al. [4] that was derived from Fiske’s work on scientist stereotypes [5]]

- Narcissism related terms: Self-focused (R), Vain (R), Modest
- Warmth related terms: Altruistic, Sincere, Honest, Dishonest (R), Warm, Helpful, Sociable, Ethical, Unethical (R), Likeable, Friendly, Trustworthy
- Competence related terms: Competent, Confident, Competitive, Intelligent
- Physically attractive

The spokesperson in the video is… [order of statements presented is randomly decided, 7-point semantic differentials] [modified from “When it comes to genetic science, direct-to-consumer genetic testing companies are…” from Miller et al. [6] which was adapted from McCroskey et al. [7] [trustworthiness: 1-5; expertise: 6-8]

1. Honest - Dishonest
2. Good - Bad
3. Valuable - Worthless
4. Selfish - Unselfish (R)
5. Sinful - Virtuous (R)
6. An expert - Inexpert
7. Intelligent - Unintelligent
8. Qualified - Unqualified

I perceive the video I watched to be… [order of statements presented is randomly decided, 7-point semantic differentials] [semantic differentials from Kim & Cameron’s [8] Corporate Message Credibility based on Ohanian [9]] [trustworthiness: 1-6]

1. Unbiased - Biased
2. Accurate - Inaccurate
3. Believable - Unbelievable
4. Convincing - Unconvincing
5. Trustworthy - Untrustworthy
6. Telling the whole story - Not telling the whole story

Please state the degree to which you agree or disagree with the following statements. [order of statements presented is randomly decided, 7-point scale from strongly disagree to strongly agree] [interest/enjoyment - subset of the intrinsic motivation inventory by Ryan [10]]

- I enjoyed this video very much.
- This video was fun to watch.
- I thought this was a boring video. (R)
- This video did not hold my attention at all. (R)
- I would describe this video as very interesting.
- I thought this video was quite enjoyable.
- While I was watching this video, I was thinking about how much I enjoyed it.
- Please select “Neither agree nor disagree”. - quality control question, failure to answer correctly terminates survey

Please state the degree to which you agree or disagree with the following statements. [order of statements presented is randomly decided, 7-point scale from strongly disagree to strongly agree] [attitude toward research (1-3) and funding (4-6) of science depicted in video]

1. Even if it brings no immediate benefits, scientific research, like this study, is necessary and important.
2. Scientific research, like this, that describes new species is scientifically important.
3. Scientific research, like this, done for the sole purpose of advancing the frontiers of knowledge benefits society.
4. Museum natural history collections, such as featured in this video, are important.
5. Museum natural history collections, such as featured in this video, should receive public taxpayer support.
6. Scientists who work in natural history collections, such as featured in this video, should receive public taxpayer support.
7. Please select “Somewhat disagree”. - quality control question, failure to answer correctly terminates survey

Please take a couple of minutes to write in the space provided any thoughts you had pertaining to the content in the video you watched. The thoughts you list could deal with your impressions or any other thoughts that might have crossed your mind. [long text response, requested required]

Demographic Questions:

How do you describe yourself? [dropdown menu]

- Male
- Female
- Do not identify as male or female
- Prefer not to say

Are you of Hispanic or Latino origin or descent? [multiple choice]

- Yes
- No

What is your ethnicity? Select as many as apply (Categories and descriptions from census.gov). [multiple choice, multiple answer allowed]

- Asian/Asian-American (A person having origins in any of the original peoples of the Far East, Southeast Asia, or the Indian subcontinent including, for example, Cambodia, China, India, Japan, Korea, Malaysia, Pakistan, the Philippine Islands, Thailand, and Vietnam.)
- Black/African American (A person having origins in any of the Black racial groups of Africa)
- Native American/American Indian (A person having origins in any of the original peoples of North and South America (including Central America) and who maintains tribal affiliation or community attachment.)
- Native Hawaiian or Other Pacific Islander (A person having origins in any of the original peoples of Hawaii, Guam, Samoa, or other Pacific Islands.)
- White (A person having origins in any of the original peoples of Europe, the Middle East, or North Africa.)
- Other

What political party do you identify with most? [multiple choice]

- Democratic party
- Republican party
- Other [and a blank to type in]

Which ideology do you align most with? [multiple choice]

- Very conservative
- Conservative
- Lean conservative
- Lean liberal
- Liberal
- Very liberal

What is the highest level of education you have completed? [multiple choice]

- Some high school or less
- High school diploma or GED
- Some college
- Trade/vocational training
- Associate’s degree
- Bachelor’s degree
- Master’s degree
- Professional degree
- Doctoral degree

Is your degree in a STEM field? [multiple choice]

- Yes
- No

What is your annual household income before taxes (gross)? [multiple choice]

- Less than $25,000
- $25,000 to $34,999
- $35,000 to $54,999
- $55,000 to $74,999
- $75,000 to $99,999
- $100,000 to $149,999
- $150,000 or more

Survey Submitted and Debrief Message:

“Your response has now been recorded.

Debrief Statement: The study you just completed was designed to examine how the spokesperson and their relationship to scientific research influences an individual's perception of that science. While the ant species discussed in the video you viewed are real species, some of the details regarding their discovery and who made that discovery were fabricated by the researchers specifically for this study and are not factual.

Thank you for your participation and for not discussing the contents of this study with others. If you have any questions about this study, please contact Dr. Adrian Smith at aasmith7@ncsu.edu.”

**Supplemental Analyses and Results**

**Demographic variables**

Our respondents varied in age, gender identity, ethnicity, Hispanic descent, level of education, religion, ideology, and annual gross household income. The breakdown of our participants by treatment group (scientist, third-party spokesperson, no on-screen spokesperson) are summarized in Table A.

**Table A. Summary of demographic breakdown of respondents across treatments.** Values represent number of responses for categorical variables and mean (± standard deviation) for continuous variables.

|  | **Treatments** | | |  |
| --- | --- | --- | --- | --- |
| **Demographic variable** | **Scientist** | **Third-party** | **No on-screen spokesperson** | **Overall** |
| Participant age (mean ± standard deviation) | 48.49 ± 17.12 | 44.68 ± 16.55 | 48.56 ± 16.81 | 46.97 ± 16.90 |
| Gender identity   - Male - Female - Do not identify as male or female | - 73 - 133 - 0 | - 70 - 135 - 2 | - 38 - 64 - 0 | - 181 - 332 - 2 |
| Hispanic or Latino origin or descent   - Yes - No | - 15 - 191 | - 22 - 185 | - 13 - 89 | - 50 - 465 |
| Ethnicity   - Asian American - African American - Native American - Pacific Islander - White - Other - Multiple responses (choose more than one above) | - 9 - 26 - 1 - 1 - 163 - 3 - 3 | - 4 - 21 - 5 - 0 - 164 - 8 - 5 | - 2 - 3 - 2 - 0 - 85 - 6 - 4 | - 15 - 50 - 8 - 1 - 412 - 17 - 12 |
| Political party   - Democratic - Republican - Other | - 96 - 80 - 30 | - 100 - 65 - 42 | - 50 - 39 - 13 | - 246 - 184 - 85 |
| Ideology   - Very conservative - Conservative - Lean conservative - Lean liberal - Liberal - Very liberal | - 23 - 39 - 54 - 40 - 26 - 24 | - 16 - 31 - 45 - 44 - 42 - 29 | - 13 - 13 - 28 - 26 - 12 - 10 | - 52 - 83 - 127 - 110 - 80 - 63 |
| Education level   - Some high school or less - High school diploma or GED - Some college - Trade/vocational training - Associate’s degree - Bachelor’s degree - Master’s degree - Professional degree - Doctoral degree | - 4 - 42 - 52 - 7 - 18 - 54 - 22 - 3 - 4 | - 5 - 44 - 50 - 7 - 18 - 50 - 26 - 4 - 3 | - 3 - 18 - 26 - 1 - 14 - 24 - 14 - 1 - 1 | - 12 - 104 - 128 - 15 - 50 - 128 - 62 - 8 - 8 |
| STEM degree   - Yes - No | - 29 - -177 | - 24 - 183 | - 9 - -93 | - 62 - 453 |
| Gross annual household income   - Less than $25,000 - $25,000 to $34,999 - $35,000 to $54,999 - $55,000 to $74,999 - $75,000 to $99,999 - $100,000 to $149,999 - $150,000 or more | - 57 - 23 - 38 - 30 - 22 - 17 - 19 | - 51 - 25 - 33 - 38 - 24 - 18 - 18 | - 29 - 9 - 23 - 16 - 14 - 8 - 3 | - 137 - 57 - 94 - 84 - 60 - 43 - 40 |

**Distribution across treatments**

To determine if demographic variables differed among our treatment groups with conducted either a one-way ANOVA (for continuous variables) or chi-square tests (for categorical variables) for each of the self-reported demographics. For some of these analyses, we rescaled the demographic variables into binary variables to facilitate incorporation as covariates in our ANCOVA models (see main text) if variables were significantly different across treatments. Of all the demographic variables tested, only age (continuous) and ideology (rescaled into a binary conservative vs. liberal variable) were significant across treatments. Ethnicity was rescaled to identifying as white versus other. In this case, other represented any response other than white or a response that indicated multiple ethnicities. A summary of the demographic variable, rescaled values, test run, and whether significant across treatments is including in Table B.

**Table B. Summary of test statistics determining if demographic variables differed across treatments (scientist, third-party, no on-screen spokesperson).** Categorical variables were rescaled into binary variables prior to completing analyses. Significant *P*-values are in bold.

| **Demographic variable** | **Category** | **Test run** | **Test Statistic** | **Degrees of Freedom** | ***P*-value** |
| --- | --- | --- | --- | --- | --- |
| Participant age | Continuous | ANOVA | 3.203 | 2, 512 | **0.041** |
| Gender identity | Categorical (binary: male vs. female) | Chi-square | 0.292 | 2 | 0.864 |
| Hispanic or Latino origin or descent | Categorical (binary: yes vs. no) | Chi-square | 2.657 | 2 | 0.265 |
| Ethnicity | Categorical (binary: white vs. other) | Chi-square | 0.884 | 2 | 0.643 |
| Political party | Categorical (not rescaled) | Chi-square | 5.067 | 4 | 0.281 |
| Ideology | Categorical (binary: conservative vs. liberal) | Chi-square | 6.035 | 2 | **0.049** |
| Education level | Categorical (binary: has college degree vs. not) | Chi-square | 0.534 | 2 | 0.766 |
| STEM degree | Categorical (binary: yes vs. no) | Chi-square | 1.843 | 2 | 0.398 |
| Gross annual household income | Categorical (binary: below $55k vs. above $55k) | Chi-square | 1.673 | 2 | 0.433 |

**Additional analyses**

**General attitude towards science and deference to scientific authority**

We ran individual ANCOVAs with treatment as fixed effect and both participant age (continuous) and ideology (binary: conservative vs. liberal) as covariates to determine if there was an effect of treatment on participants general attitude towards science or deference to scientific authority. There was no effect of treatment on general attitude towards science or on deference to scientific authority (Table C).

**Other perceptions of scientists in general**

In addition to measuring the warmth and competence of scientists in general, we also asked respondents to assess how well the following traits were characteristics of scientists in general – self-focused, vain, modest, and physically attractive. Out of these one-item traits, only whether respondents viewed scientists in general as being vain was significantly different among treatments (*F*_2,510_ = 4.34, *P* = 0.014; Table C) when false discovery rates were not taken into account. In this case, respondents who viewed a spokesperson on screen, irrespective of whether that spokesperson identified as the scientist who conducted the work or a third-party individual, rated scientists in general as being moderately less vain than when no spokesperson was on-screen to explain the science story (scientist vs. third-party: *P* = 1.00; scientist vs. no on-screen spokesperson: *P* = 0.026; third-party vs. no on-screen spokesperson: *P* = 0.019). These results disappear when false discovery rates are taken into account and therefore should be viewed with caution. Additionally, scientists in general were always viewed as being slightly self-focused, and unattractive, and only moderately modest irrespective of which treatment was viewed.

**Table C. ANCOVA and post-hoc analyses of the effects of treatment (scientist vs. third-party vs. no on-screen spokesperson) on outcome measures. Adjusted *P* values account for false discovery rates.**

|  | **Estimated marginal mean (standard error)** | | |  |  |  |
| --- | --- | --- | --- | --- | --- | --- |
|  | **Scientist** | **Third-party** | **No on-screen spokesperson** | **F(d1,d2)** | ***P* value (adjusted *P* value)** | **Partial eta squared** |
| **General attitude towards science** | 5.81 (0.07) | 5.77 (0.07) | 5.75 (0.09) | 0.15 (2,510) | 0.863 (0.935) | 0.001 |
| **Deference to scientific authority** | 5.12 (0.08) | 5.07 (0.08) | 5.03 (0.11) | 0.30 (2,510) | 0.738 (0.935) | 0.001 |
| **Scientist** |  |  |  |  |  |  |
| Self-focused (reverse coded) | 2.89 (0.08) | 2.93 (0.08) | 2.83 (0.12) | 0.22 (2, 510) | 0.804 (0.935) | 0.001 |
| Vain (reverse coded) | 3.57 (0.07)_a_ | 3.58 (0.07)_a_ | 3.23 (0.10)_b_ | 4.34 (2, 510) | 0.014 (0.0653) | 0.017 |
| Modest | 3.32 (0.07) | 3.25 (0.07) | 3.14 (0.10) | 1.30 (2, 510) | 0.275 (0.679) | 0.005 |
| Physically attractive | 2.81 (0.06) | 2.88 (0.06) | 2.70 (0.09) | 1.45 (2, 510) | 0.237 (0.679) | 0.006 |

Higher scores represent more positive perceptions of scientists. Lowercase subscript letters denote significant post-hoc pairwise comparisons with Bonferroni adjustments at or below the *P* < 0.05. These results should be examined critically as these post-hoc comparisons are conducted when main effects were significant based on unadjusted *P* values.

**Influence of spokesperson gender identity on audience perceptions**

Additionally, we explored how differences in spokesperson gender identity within each of the treatments influenced public perceptions. However, as there was only one male-presenting and one female-presenting spokesperson, there are many potential confounding factors that could influence any differences found in perceptions. For example, our spokespeople varied in age, Hispanic descent, and, though they used the same script to present the science story, each had their own unique on-screen presence and presentation style. As such, results presented here should be interpreted with caution. Additionally, the main analyses of interest are across spokesperson condition rather than individual videos and splitting our sample size across videos reduces the power of our comparisons.

As with our previous analyses by treatment, we first assessed whether demographic variables differed significantly across videos (summary in Tables D and E). Of the demographic variables tested, only participant age (continuous) was significant while ideology (binary: conservative vs. liberal) was marginally significant). These two covariates were already demonstrated to have a significant yet extremely weak correlation (r = -0.09, *P* = 0.042) therefore we use these covariates in our individual ANCOVA models for each outcome measure of interest.

**Table D. Summary of demographic breakdown of respondents across videos.** Categorical variables were rescaled into binary variables prior to completing analyses. Significant *P*-values are in bold.

|  | **Treatments** | | | | |
| --- | --- | --- | --- | --- | --- |
| **Demographic variable** | **Scientist male** | **Scientist female** | **Third-party male** | **Third-party female** | **No on-screen spokesperson** |
| Participant age (mean ± standard deviation) | 47.39 ± 17.03 | 49.56 ± 17.23 | 47.22 ± 16.12 | 42.12 ± 16.65 | 48.56 ± 16.81 |
| Gender identity   - Male - Female - Do not identify as male or female | - 40 - 62 - 0 | - 33 - 71 - -0 | - 37 - 66 - 1 | - 33 - 69 - 1 | - 38 - 64 - 0 |
| Hispanic or Latino origin or descent   - Yes - No | - 6 - 96 | - 9 - 95 | - 14 - 90 | - 8 - 95 | - 13 - 89 |
| Ethnicity   - Asian American - African American - Native American - Pacific Islander - White - Other - Multiple responses (choose more than one above) | - 5 - 17 - 0 - 1 - 77 - 2 - 0 | - 4 - 9 - 1 - 0 - 86 - 1 - 3 | - 1 - 10 - 2 - 0 - 83 - 6 - 2 | - 3 - 11 - 3 - 0 - 81 - 2 - 3 | - 2 - 3 - 2 - 0 - 85 - 6 - 4 |
| Political party   - Democratic - Republican - Other | - 52 - 38 - 12 | - 44 - 42 - 18 | - 44 - 37 - 23 | - 56 - 28 - 19 | - 50 - 39 - 13 |
| Ideology   - Very conservative - Conservative - Lean conservative - Lean liberal - Liberal - Very liberal | - 12 - 23 - 18 - 27 - 10 - 12 | - 11 - 16 - 36 - 13 - 16 - 12 | - 8 - 20 - 23 - 23 - 21 - 9 | - 8 - 11 - 22 - 21 - 21 - 20 | - 13 - 13 - 28 - 26 - 12 - 10 |
| Education level   - Some high school or less - High school diploma or GED - Some college - Trade/vocational training - Associate’s degree - Bachelor’s degree - Master’s degree - Professional degree - Doctoral degree | - 3 - 17 - 23 - 2 - 13 - 31 - 11 - 2 - 0 | - 1 - 25 - 29 - 5 - 5 - 23 - 11 - 1 - 4 | - 2 - 20 - 28 - 3 - 6 - 27 - 15 - 1 - 2 | - 3 - 24 - 22 - 4 - 12 - 23 - 11 - 3 - 1 | - 3 - 18 - 26 - 1 - 14 - 24 - 14 - 1 - 1 |
| STEM degree   - Yes - No | - 12 - 90 | - 17 - 87 | - 12 - 92 | - 12 - 91 | - 9 - 93 |
| Gross annual household income   - Less than $25,000 - $25,000 to $34,999 - $35,000 to $54,999 - $55,000 to $74,999 - $75,000 to $99,999 - $100,000 to $149,999 - $150,000 or more | - 24 - 7 - 29 - 14 - 14 - 6 - 8 | - 33 - 16 - 9 - 16 - 8 - 11 - 11 | - 25 - 15 - 16 - 18 - 8 - 12 - 10 | - 26 - 10 - 17 - 20 - 16 - 6 - 8 | - 29 - 9 - 23 - 16 - 14 - 8 - 3 |

**Table E. Summary of test statistics determining if demographic variables differed across videos (scientist male, scientist female, third-party male, third-party female, no on-screen spokesperson).** Categorical variables were rescaled into binary variables prior to completing analyses. Significant *P*-values are in bold and marginally significant *P*-values are in italics.

| **Demographic variable** | **Category** | **Test run** | **Test Statistic** | **Degrees of Freedom** | ***P*-value** |
| --- | --- | --- | --- | --- | --- |
| Participant age | Continuous | ANOVA | 3.026 | 4, 510 | **0.017** |
| Gender identity | Categorical (binary: male vs. female) | Chi-square | 1.841 | 4 | 0.765 |
| Hispanic or Latino origin or descent | Categorical (binary: yes vs. no) | Chi-square | 5.022 | 4 | 0.285 |
| Ethnicity | Categorical (binary: white vs. other) | Chi-square | 2.597 | 4 | 0.627 |
| Political party | Categorical (not rescaled) | Chi-square | 9.995 | 8 | 0.265 |
| Ideology | Categorical (binary: conservative vs. liberal) | Chi-square | 9.329 | 4 | *0.053* |
| Education level | Categorical (binary: has college degree vs. not) | Chi-square | 4.335 | 4 | 0.363 |
| STEM degree | Categorical (binary: yes vs. no) | Chi-square | 2.864 | 4 | 0.581 |
| Gross annual household income | Categorical (binary: below $55k vs. above $55k) | Chi-square | 1.988 | 4 | 0.738 |

The ANCOVA results for each outcome measure are summarized in Table E. The video the respondents viewed has a significant effect on spokesperson expertise (*F*_2,508_ = 3.60, *P* = 0.007; Tables F,G) and spokesperson trustworthiness (*F*_2,508_ = 2.86, *P* < 0.05; Tables F,G) when false discovery rates were not taken into account. None of the other outcome measures were significantly affected by which video was viewed. When false discovery rates were not taken into account, participants who viewed a video presented in the first person by a female scientist perceived that spokesperson as having more expertise than the video narrated by a third-party male spokesperson or when there was no on-screen spokesperson with the videos narrated either by the male scientist or third-party female having intermediate spokesperson expertise. Participants who viewed a video presented by the female scientist perceived that spokesperson as being more trustworthy than viewing a video where no spokesperson appeared on screen, with all other videos having an intermediate level of spokesperson trustworthiness. However, when false discovery rates are taken into account these significant effects go away.

**Table F. ANCOVA and post-hoc analyses of the effects of video stimulus (scientist male vs. scientist female vs. third-party male vs. third-party female vs. no on-screen spokesperson) on outcome measures. Adjusted *P* values account for false discovery rates.**

|  | **Estimated marginal mean (standard error)** | | | | |  |  |  |
| --- | --- | --- | --- | --- | --- | --- | --- | --- |
|  | **Scientist male** | **Scientist female** | **Third-party male** | **Third-party female** | **No on-screen spokesperson** | **F(d1,d2)** | ***P* value (adjusted *P* value)** | **Partial eta squared** |
| **Spokesperson** |  |  |  |  |  |  |  |  |
| Expertise | 5.92 (0.12)_ab_ | 6.16 (0.12)_a_ | 5.63 (0.12)_b_ | 5.70 (0.12)_ab_ | 5.63 (0.12)_b_ | 3.60 (4,508) | 0.007 (0.098) | 0.028 |
| Trustworthiness | 5.73 (0.11)_ab_ | 5.89 (0.11)_a_ | 5.59 (0.11)_ab_ | 5.64 (0.11)_ab_ | 5.40 (0.11)_b_ | 2.86 (4,508) | 0.023 (0.161) | 0.022 |
| **Stimulus** |  |  |  |  |  |  |  |  |
| Trustworthiness | 5.92 (0.12) | 6.01 (0.12) | 5.85 (0.12) | 5.80 (0.12) | 5.84 (0.12) | 0.48 (4,508) | 0.747 (0.974) | 0.004 |
| Enjoyment | 4.40 (0.16) | 4.54 (0.15) | 4.43 (0.15) | 4.47 (0.16) | 4.53 (0.16) | 0.17 (4,508) | 0.955 (0.974) | 0.001 |
| **Scientists** |  |  |  |  |  |  |  |  |
| Competence | 4.25 (0.06) | 4.22 (0.06) | 4.19 (0.06) | 4.23 (0.06) | 4.21 (0.06) | 0.12 (4,508) | 0.974 (0.974) | 0.001 |
| Warmth | 3.66 (0.07) | 3.62 (0.07) | 3.64 (0.07) | 3.65 (0.07) | 3.52 (0.07) | 0.68 (4,508) | 0.606 (0.943) | 0.005 |
| Self-focused (reverse coded) | 2.72 (0.12) | 3.06 (0.12) | 2.94 (0.12) | 2.91 (0.12) | 2.83 (0.12) | 1.13 (4,508) | 0.338 (0.789) | 0.009 |
| Vain (reverse coded) | 3.58 (0.10) | 3.56 (0.10) | 3.58 (0.10) | 3.59 (0.11) | 3.23 (0.10) | 2.17 (4,508) | 0.071 (0.331) | 0.017 |
| Modest | 3.33 (0.10) | 3.32 (0.10) | 3.20 (0.10) | 3.30 (0.10) | 3.14 (0.10) | 0.78 (4,508) | 0.542 (0.943) | 0.006 |
| Physically attractive | 2.89 (0.09) | 2.71 (0.09) | 2.78 (0.08) | 2.97 (0.09) | 2.70 (0.09) | 1.88 (4,508) | 0.112 (0.392) | 0.015 |
| **Attitudes** |  |  |  |  |  |  |  |  |
| Research | 5.62 (0.11) | 5.68 (0.11) | 5.55 (0.10) | 5.58 (0.11) | 5.60 (0.11) | 0.23 (4,508) | 0.921 (0.974) | 0.002 |
| Funding | 5.04 (0.13) | 5.30 (0.13) | 5.19 (0.13) | 5.11 (0.13) | 5.02 (0.13) | 0.78 (4,508) | 0.536 (0.943) | 0.006 |
| **General attitude towards science** | 5.86 (0.09) | 5.76 (0.09) | 5.82 (0.09) | 5.72 (0.09) | 5.75 (0.09) | 0.36 (4,508) | 0.837 (0.974) | 0.003 |
| **Deference to scientific authority** | 5.27 (0.11) | 4.98 (0.11) | 5.13 (0.11) | 5.01 (0.11) | 5.02 (0.11) | 1.25 (4,508) | 0.290 (0.789) | 0.01 |

Higher scores represent more positive perceptions. Lowercase subscript letters denote significant post-hoc pairwise comparisons with Bonferroni adjustments at or below the *P* < 0.05. These results should be examined critically as these post-hoc comparisons are conducted when main effects were significant based on unadjusted *P* values.

**Table G. Tukey post-hoc tests with Bonferroni adjustments when false discovery rates are not taken into account.**

|  |  | ***P* values** | |
| --- | --- | --- | --- |
| **First comparison video** | **Second comparison video** | **Spokesperson expertise** | **Spokesperson trustworthiness** |
| Female scientist | Male scientist | 1.00 | 1.00 |
| Female scientist | Third-party female | 0.076 | 0.921 |
| Female scientist | Third-party male | 0.021 | 0.443 |
| Female scientist | No on-screen spokesperson | 0.020 | 0.012 |
| Male scientist | Third-party female | 1.00 | 1.00 |
| Male scientist | Third-party male | 0.953 | 1.00 |
| Male scientist | No on-screen spokesperson | 0.926 | 0.328 |
| Third-party female | Third-party male | 1.00 | 1.00 |
| Third-party female | No on-screen spokesperson | 1.00 | 1.00 |
| Third-party male | No on-screen spokesperson | 1.00 | 1.00 |

**Supplemental References**

1. MacGown JA, Boudinot B, Deyrup M, Sorger DM. A review of the Nearctic *Odontomachus* (Hymenoptera: Formicidae: Ponerinae) with a treatment of the males. Zootaxa. 2014;3802: 515. doi:10.11646/zootaxa.3802.4.6

2. National Science Board. Science and engineering indicators 2018. 2018 [cited 31 Mar 2021]. Available: https://www.nsf.gov/statistics/2018/nsb20181/

3. Brossard D, Nisbet MC. Deference to scientific authority among a low information public: Understanding U.S. opinion on agricultural biotechnology. Int J Public Opin Res. 2006;19: 24–52. doi:10.1093/ijpor/edl003

4. Jarreau PB, Cancellare IA, Carmichael BJ, Porter L, Toker D, Yammine SZ. Using selfies to challenge public stereotypes of scientists. PLOS ONE. 2019;14: e0216625. doi:10.1371/journal.pone.0216625

5. Fiske ST, Dupree C. Gaining trust as well as respect in communicating to motivated audiences about science topics. PNAS. 2014;111: 13593–13597. doi:10.1073/pnas.1317505111

6. Miller CH, Lane LT, Deatrick LM, Young AM, Potts KA. Psychological reactance and promotional health messages: The effects of controlling language, lexical concreteness, and the restoration of freedom. Hum Commun Res. 2007;33: 219–240. doi:10.1111/j.1468-2958.2007.00297.x

7. McCroskey JC, Jensen T, Todd C. The generalizability of source credibility scales for public figures. Chicago, Illinois; 1972. Available: https://eric.ed.gov/?id=ED074560

8. Kim HJ, Cameron GT. Emotions matter in crisis: The role of anger and sadness in the publics’ response to crisis news framing and corporate crisis response. Commun Res. 2011;38: 826–855. doi:10.1177/0093650210385813

9. Ohanian R. Construction and validation of a scale to measure celebrity endorsers’ perceived expertise, trustworthiness, and attractiveness. J Advert. 1990;19: 39–52. doi:10.1080/00913367.1990.10673191

10. Ryan R. Control and information in the intrapersonal sphere: An extension of cognitive evaluation theory. J Pers Soc Psychol. 1982;43: 450–461. doi:10.1037/002-3514.43.3.450
